# Inflammatory myopathy following coronavirus disease 2019 vaccination: A systematic review

**DOI:** 10.3389/fpubh.2022.1007637

**Published:** 2022-10-21

**Authors:** Yukang Ding, Yongpeng Ge

**Affiliations:** ^1^Department of Rheumatology, The Affiliated Hospital of Jiangxi University of Traditional Chinese Medicine, Nanchang, China; ^2^Department of Rheumatology, Key Laboratory of Myositis, China-Japan Friendship Hospital, Beijing, China

**Keywords:** inflammatory myopathy, dermatomyositis, coronavirus disease 2019 vaccine, SARS-CoV-2 vaccine, myositis specific autoantibodies

## Abstract

**Introduction:**

Reports of unexpected side effects have accompanied the vaccination of larger proportions of the population against coronavirus disease 2019 (COVID-19), including a few cases of inflammatory myopathy (IM). In a bid to improve understanding of the clinical course of vaccine complications, a systematic review of reported cases of IM following COVID-19 vaccination has been conducted.

**Methods:**

The PRISMA guideline 2020 was followed. Two independent investigators systematically searched PubMed and Embase to identify relevant studies published up to July 2022, using the following keywords: COVID-19 Vaccine, inflammatory myositis. The Joanna Briggs Institute critical appraisal tools were used for the risk of bias.

**Results:**

A total of 24 articles presenting clinical features of 37 patients with IM following COVID-19 vaccine were identified. Female patients composed 59.5% of cases and 82.4% had been vaccinated with BNT162b2 or ChAdOx1. Onset of symptoms occurred within 2 weeks of the first or second vaccine dose in 29 (85.3%) patients and included muscular weakness in 54.1% and skin rash in 71.4% of patients. Myositis specific autoantibodies (MSAs) and myositis associated autoantibodies (MAAs) were reported in 28 patients. Specific clinical subtypes of myositis, reported in 27 patients, included 22 (81.5%) cases of dermatomyositis (DM) and 3 (11.1%) cases of immune-mediated necrotizing myopathy (IMNM). Following treatment, 32 (86.5%) patients showed improvement on follow-up.

**Conclusion:**

COVID-19 vaccine may induce various clinical myositis subtypes and related antibodies. Muscular weakness was the most common presenting symptom. Clinicians should be aware of this unexpected adverse event following COVID-19 vaccination and arrange for appropriate management.

**Systematic review registration:**

INPLASY https://inplasy.com/inplasy-2022-9-0084/ [INPLASY202290084].

## Introduction

Inflammatory myopathy (IM) belongs to a rare group of clinically heterogeneous, autoimmune inflammatory disorders characterized by muscular weakness and multi-system involvement, including lung and skin. Dermatomyositis (DM), anti-synthetase syndrome (ASS), immune-mediated necrotizing myopathy (IMNM), polymyositis (PM) and inclusion body myositis (IBM) have long been recognized as distinct subtypes of IM (1–3). The pathogenesis of IM is complicated and poorly understood but an autoimmune response is known to be involved. Vaccines exert a potent stimulatory effect on the immune system and have the potential to induce or exacerbate serologic findings or clinical autoimmune diseases. The pandemic caused by coronavirus disease 2019 (COVID-19) had a devastating and worldwide impact on public health, social life and the economy (4–7). The development of vaccines which successfully prevent severe illness from SARS-CoV-2 infection are considered one of the effective solutions (8).

Safety concerns have surrounded the vaccines since their development, with common adverse effects including local reaction at the site of injection and diverse non-specific flu-like symptoms (9). Most symptoms occur soon after vaccination and resolve within a short period but some serious events such as myopericarditis and cerebral venous thrombosis post COVID-19 vaccination had been reported (10–12). Meanwhile, some rare cases of vaccine-associated IMs have been reported (13–36). The current study systematically reviewed IM cases reported post-COVID-19 vaccination to date. Clinical and laboratory features are described and therapy and prognosis discussed.

## Methods

### Protocol and registration

We registered our study on INPLASY website (Register number INPLASY202290084) and followed the Preferred Reporting Items for Systematic Reviews and Meta-Analyses (PRISMA) 2020 statement (37).

### Eligibility criteria

The study was undertaken to systematically review IM patients followed with COVID-19 vaccination. A PICOS acronym was used to formulate the questions for this study: (1) participants (patients with IMs), (2) intervention or exposition (COVID-19 vaccination), (3) comparison or control (not applicable), (4) outcomes measures (time, dose and type of COVID-19 vaccination), (5) types of studies included (case reports and series of cases).

We set the exclusion criteria as follows: (1) studies that described non-inflammatory myopathy, (2) reviews of the literature, personal opinions and conference abstracts, (3) full articles not found, (4) duplicate cases from other studies, (5) suspected/ probable cases of SARS-CoV-2 infection.

### Search strategy

Two independent investigators systematically searched PubMed and Embase to identify relevant studies published up to July 2022. We searched Medical Subject Headings terms and keywords in multiple combinations, including COVID-19 or SARS-CoV-2 vaccine, dermatomyositis, myositis or inflammatory myopathy.

### Data collection

Two authors evaluated abstracts and titles of each article and a full-text review was performed as relevant considering all inclusion and exclusion criteria. Another rheumatologist reviewed all articles where there was no consensus in the initial evaluation to determine which articles would be included. After selection and evaluation, data from the included studies were extracted, including study authors and year, publications, study design, and basic information of the patients.

### Risk of bias

The risk of bias for each included study was assessed using the Joanna Briggs Institute Critical Appraisal checklist for case reports and case series. Disagreements were solved between the two investigators by consensus or by another independent investigator.

## Results

### Study selection and general information

**Figure 1:**
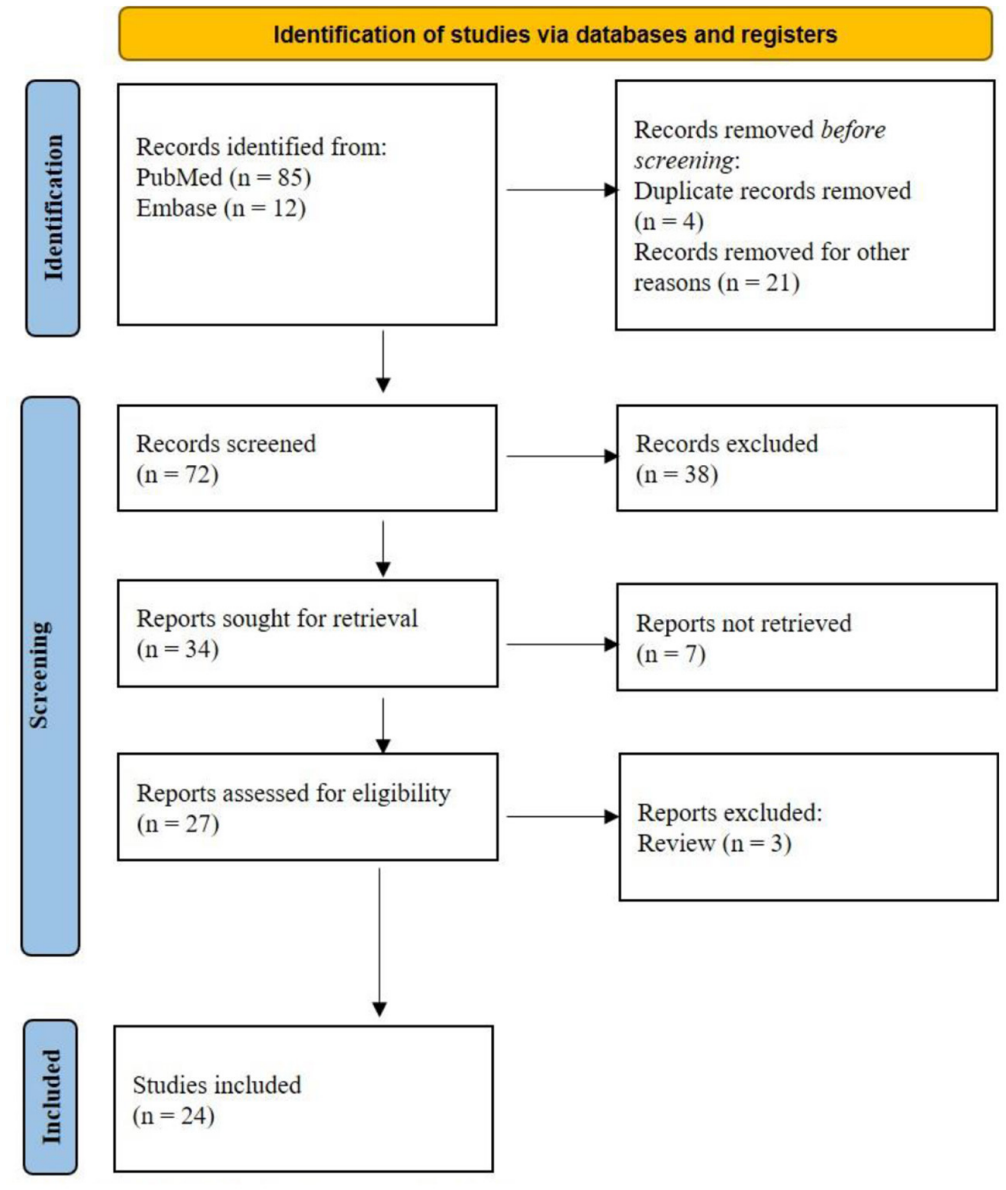
PRISMA flow diagram of study selection.

PubMed and Embase provided 97 titles and abstracts for review. Of these, a total of five articles were excluded due to duplication. After screening, 34 full articles were further assessed for eligibility. Eventually, 24 studies (10–33) containing 37 patients were selected for final analysis (Figure 1).

Twenty-two female and 15 male (ratio: 1.5: 1) patients were included with age of onset ranging from 28 to 87 years (median: 56 years). Twenty-four (68.6%) patients were over 50 years old (see Table 1).

**Table 1:** Clinical and laboratory findings of IM patients following COVID-19 vaccination.

**References**	**Age/sex**	**Vaccine**	**Latency**	**Skin rash**	**Musculoskeletal tissues**	**CK (IU/L)**	**MSAs/ MAAs**	**Treatment and outcome**
Tan et al. (13)	54/M	CoronaVac	One week after 2nd dose	No	Myalgia, muscle weakness	27,000	SRP	PSL, IVIG; improvement
Kim et al. (14)	30/M	BNT162b2	6 days after 2nd dose	Erythematous purpuric skin rash	Myalgia, muscle weakness	4,778	No	GC, AZA, TAC; improvement
Gupta et al. (15)	46/F	ChAdOx1	One week after 2nd dose	Mechanic's hands	Arthralgia, myalgia, muscle weakness	1,560	Jo-1	PSL, MTX, MMF; improvement
Wu et al. (16)	77/F	BNT162b2	5 days after 1st dose	Violaceous, scaly plaques on the neck and chest	Myalgia, muscle weakness	4,476	TIF1γ	MP, IVIG; improvement
Farooq et al. (17)	62/M	ChAdOx1	2 months later after 2nd dose	No	Myalgia, muscle weakness	4,053	NA	GC; improvement
Theodorou et al. (18)	56/F	NA	8 days after 2nd dose	No	Arthralgia, myalgia, muscle weakness	Elevated	NA	NSAIDs; improvement
Gouda et al. (19)	43/F	BNT162b2	10 days after 2nd dose	Erythematous rash all over face, trunk, and hands	Myalgia	3,358	RNP	PSL, MMF, HCQ; improvement
Venkateswaran et al. (20)	43/M	mRNA-1273	One day after 1st dose	Painful rash over the cheeks, scalp, back and chest	Muscle weakness	NA	No	PSL, IVIG, HCQ; improvement
Camargo et al. (21)	76/F	BNT162b2	One day after 2nd dose	Holster sign in both tights and the “V” sign	Myalgia, muscle weakness	3,368	Mi-2	GC, MTX; improvement
Lee et al. (22)	53/M	BNT162b2	2 weeks after 2nd dose	Anterior chest, deltoids, shoulders, and around the frontal hairline	Muscle weakness	14,659	NXP2	MP, IVIG, RTX; improvement
Dodig et al. (23)	55/F	BNT162b2	One day after 1st dose, worsening 1 day after 2nd dose	No	Muscle weakness	7,967	SRP	PSL, IVIG, MTX; improvement
Vutipongsatorn et al. (24)	55/F	BNT162b2	2 days after 1st dose	Erythematous rash on face, arms, and lower back	Muscle weakness	11,330	Mi-2/Ro-52	GC, IVIG, CYC, MMF; improvement
	72/F	BNT162b2	One day after 2nd dose	No	Muscle weakness	10,222	Fibrillarin	GC, IVIG, improvement
Maramattom et al. (25)	74/M	ChAdOx1	2 days after 1st dose	No	Arthralgia and myalgia	NA	NA	PSL, MMF; improvement
	75/F	ChAdOx1	2 days after 1st dose	No	Arthralgia and myalgia	NA	NA	PSL, MMF; improvement
	80/M	ChAdOx1	2 days after 2nd dose	No	Arthralgia and myalgia	NA	NA	PSL, MMF; improvement
Kaulen et al. (26)	F	BNT162b2	2 weeks after 1st dose	NA	Muscle weakness	Elevated	SAE1	PSL; improvement
	F	BNT162b2	2 weeks after 1st dose	NA	Muscle weakness	Elevated	Pm/Scl-75	PSL; improvement
Blaise et al. (27)	41/M	BNT162b2	10 days after 1st dose	No	Myalgia, muscle weakness	12,647	No	GC; improvement
Borio et al. (28)	62/M	NA	NA	Skin rash on face, chest, hands and legs	Arthralgia, myalgia	1,759	MDA5	MP, IVIG; fatal
Yoshida et al. (29)	81/F	BNT162b2	2 weeks after 1st dose	Bilateral eyelid oedema	Muscle weakness	3,119	TIF1γ	PSL, IVIG; improvement
	87/F	BNT162b2	One week after 1st dose	Bilateral eyelid oedema	Muscle weakness	Elevated	TIF1γ	PSL; improvement
Capassoni et al. (30)	37/F	ChAdOx1	4 days after 1st dose	Vesicular papular plaques on thighs, feet, and face	Muscle weakness	NA	Pm/Scl-75	GC; improvement
Kreuter et al. (31)	68/F	BNT162b2	8 days after 2nd dose	Violaceous papules and plaques on the face, trunk, arms and buttocks.	No	Normal	TIF1γ and SRP	GC, IVIG, RTX; improvement
Ooi et al. (32)	44/M	mRNA-1273	2 weeks after 1st dose; worsened after 2nd dose	Skin rash on face, poikilodermatous plaques over upper back	No	Normal	TIF1γ	PSL, HCQ; improvement
Gouveia et al. (33)	49/M	Janssen	10 days after unknown dose	No	Myalgia, muscle weakness	>1,300	NA	PSL; improvement
Carrasco et al. (34)	58/M	NA	4 days after unknown dose	Oral blisters, digital tip ischemia and ulceration	No	Normal	MDA5	GC, CYC, TAC, IVIG; fatal
Gonzalez et al. (35)	45/M	mRNA-1273	2 days after 2nd dose	Heliotrope rash, Gottron's papules, V-sign, Shawl sign	No	2,066	MDA5/RO-52	GC, IVIG, RTX; improvement
	58/F	ChAdOx1	7 days after 2nd dose	Gottron's sign, Auricular papules	No	Normal	MDA5	GC, MMF, HCQ, CYC, RTX, TOF, TAC; improvement
	45/F	BNT162b2	3 days after 2nd dose	Heliotrope rash, Gottron's papules, Mechanic hands	No	Normal	MDA5	ADA, PSL, RTX, TOF, PLEX; improvement
	28/F	BNT162b2	2 weeks after 2nd dose	Heliotrope rash, Gottron's sign, V-sign	Muscle weakness	Normal	MDA5/TIF1-γ	GC, HCQ, MMF; improvement
	51/F	BNT162b2	7 days after 2nd dose	Gottron's papules, Holster sign, Shawl sign	No	Normal	MDA5/RO-52	GC, CYC; improvement
	54/F	BNT162b2	2 weeks after 1st dose	Heliotrope rash, Gottron's papules, Mechanic hands, V-sign	No	Normal	MDA5/RO-52	GC, HCQ, AZA, MMF; improvement
Kitajima et al. (36)	71/F	mRNA-1273	12 weeks after 1st dose	Gottron papules, Gottron sign, nailfold abnormalities	No	63	MDA5	GC, TAC, CYC; fatal
	82/M	BNT162b2	8 weeks after 1st dose	Gottron's papules, Gottron's sign, nailfold abnormalities	No	451	MDA5	GC, TAC; fatal
	68/M	BNT162b2	6 weeks after 1st dose	Gottron's papules, nailfold abnormalities	No	40	MDA5	GC, TAC, CYC, TOF, PLEX; improvement
	59/F	BNT162b2	6 weeks after 1st dose	Gottron's papules, Gottron's sign, nailfold abnormalities	No	63	MDA5	GC, TAC, CYC; fatal

ADA, adalimumab; AZA, azathioprine; CK, creatine kinase; CYC, cyclophosphamide; F, Female, HCQ, hydroxychloroquine; IVIG, intravenous immunoglobulin; GC, glucocorticoid; M, Male; MASs, myositis specific antibodies; MAAs, myositis related antibodies; MDA5, melanoma differentiation-associated protein-5; MMF, mycophenolate mofetil; MP, methylprednisolone; MTX, methotrexate; NA, not applicable; NSAIDs, nonsteroidal anti-inflammatory drugs; NXP2, nuclear matrix protein-2; PLEX, Plasma exchange; PSL, prednisolone; RNP, ribonucleoprotein particle; RTX, rituximab; SAE, small ubiquitin-like modifier activating enzyme; SRP, signal recognition particle; TAC, tacrolimus; TIF1γ, transcriptional intermediary factor 1γ; TOF, Tofacitinib.

### COVID-19 vaccine information

COVID-19 vaccine manufacturers were reported by 21 studies including 34 patients as follows: 21 (61.8%) received Pfizer/BioNTech (BNT162b2); 7 (20.6%) Oxford-AstraZeneca (AZD1222, ChAdOx1); 4 Moderna (mRNA-1273); 1 Janssen and 1 Sinovac Biotech (CoronaVac) vaccines. Thirty-four patients reported the interval between vaccination and symptom onset with 18 (52.9%) patients developing symptoms after the first dose of COVID-19 vaccine and 16 (47.1%) after the second dose. Furthermore, 14/18 (77.8%) patients developed symptoms within 2 weeks of the first dose and 15/16 (93.8%) within 2 weeks of the second dose. Overall, 29/34 (85.3%) patients developed myositis symptoms within 2 weeks of the first or second dose of the COVID-19 vaccine.

### Clinical characteristics

The vast majority of patients (26/37; 70.3%) had muscle involvement with 13 (35.1%%) cases showing myalgia and 20 (54.1%) muscle weakness. Pharyngeal muscles were involved in four patients, causing dysarthria and dysphagia. From available data, 25 patients (71.4%) with skin symptoms, including the Heliotrope sign, Gottron's rash, “V” sign, mechanic hand and other atypical rashes. Available clinical data indicated that 12/30 (40%) patients had fever, 8 (26.7%) had arthralgia and 14 (46.7%) dyspnea. Interstitial lung disease (ILD) was reported in 13 (43.3%) patients.

**Figure 2:**
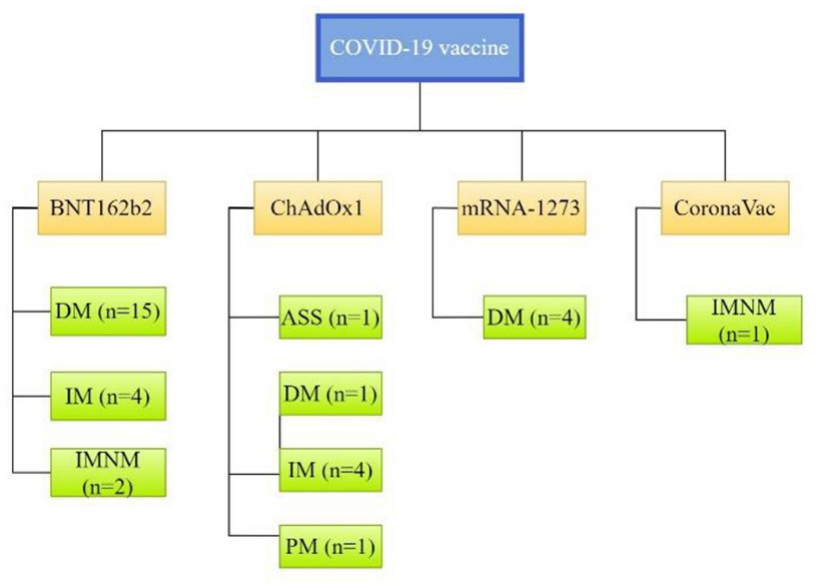
Clinical subtypes of myositis associated with individual CoV-19 vaccines. ASS, anti-synthetase syndrome; DM, dermatomyositis; IM, inflammatory myopathy; IMNM, immune-mediated necrotizing myopathy; PM, polymyositis.

T2-weighted magnetic resonance imaging (MRI) was performed for 15 patients and demonstrated increased uptake in muscle groups. Inflammatory myopathy was confirmed by muscle biopsy or skin biopsy in 18 patients. Serum CK increased significantly in 21 patients, ranging from 451 to 27,000 IU/L. Specific clinical subtypes of myositis were reported for 27 patients, 22 (81.5%) of whom had DM, 3 (11.1%) had IMNM, 1 (3.7%) had ASS, 1 (3.7%) had PM and the other 10 patients received no specific classification (see Figure 2).

**Figure 3:**
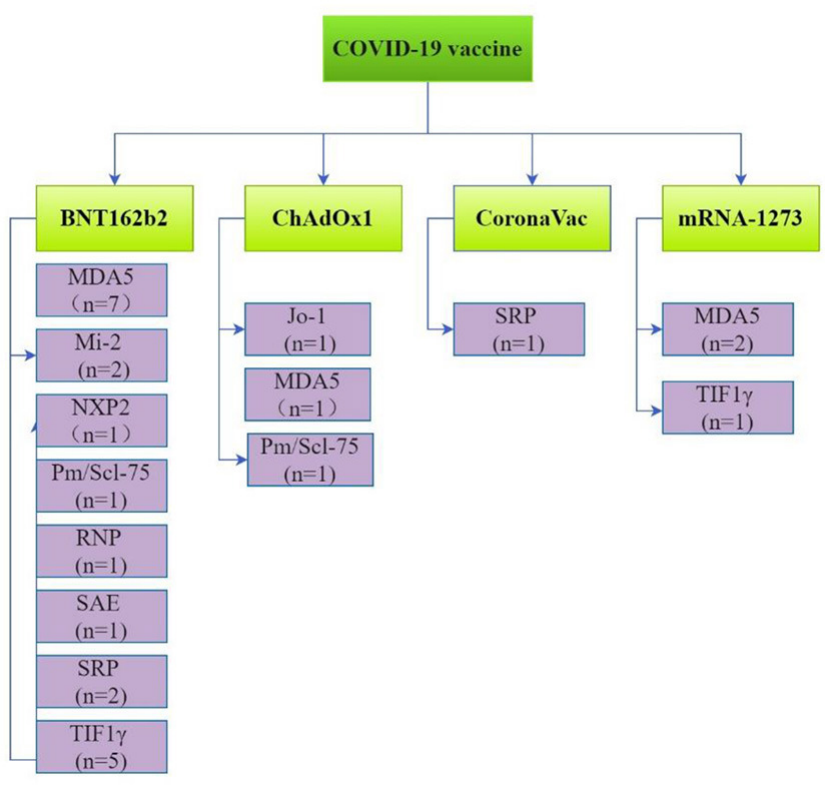
Myositis antibodies associated with individual CoV-19 vaccines.

Myositis specific antibodies (MASs) and myositis related antibodies (MAAs) were reported in 28 patients. These included antibodies against melanoma differentiation-associated protein-5 (MDA5; *n* = 11), transcriptional intermediary factor 1γ (TIF1γ; *n* = 6), signal recognition particle (SRP; *n* = 3), Mi-2 (*n* = 2), nuclear matrix protein-2 (NXP2; *n* = 1), small ubiquitin-like modifier activating enzyme (SAE; *n* = 1), Jo-1 (*n* = 1), PM/Scl-75 (*n* = 2), fibrillarin (*n* = 1) and ribonucleoprotein particle (RNP; *n* = 1; Figure 3).

### Treatment and prognosis

A range of treatments was reported by the literature under review. One patient received no immunotherapy except for non-steroidal anti-inflammatory drugs (NSAIDs). Thirty-six patients were treated with glucocorticoids, with nine receiving oral and/or intravenous glucocorticoid monotherapy and 27 patients receiving glucocorticoids combined with intravenous immunoglobulin (IVIG) or other immune agents, such as methotrexate (MTX), cyclophosphamide (CYC) or Rituximab (RTX). Thirty-two (86.5%) patients had experienced partial or complete remission by follow-up and 5 patients of MDA5-DM had poor prognosis due to rapid progression of ILD and lung infection.

### Risk of bias in included studies

High risk of bias was identified in this analysis due to the lack of description of the demographic characteristics for some patients in the case reports and case series. In addition, this review described rarity of such events; therefore, the included studies were highly heterogeneous. Although the representative sample of each study was not a cause for concern, this caused the lack of statistical data for additional analysis.

## Discussion

The current literature review indicates that COVID-19 vaccination-associated IM patients were predominantly women with a median age of 56 years. Most patients were vaccinated with Pfizer/BioNTech's (BNT162b2) and more than half reported symptoms following the first dose. Most patients had muscle weakness and a range of myositis subtypes had occurred. Most patients responded well to glucocorticoids and/or immunosuppressants.

As with other autoimmune conditions, the precise etiology of IM is unknown, although genetic and environmental factors determine susceptibility (38). The category of environmental factors may be considered to include a number of vaccines (39). Vaccine-induced autoimmunity may be due to molecular mimicry between host cell and antigen or to a direct response to vaccine adjuvants. A clear link between IMs and vaccination has yet to be found but there remain many case reports of vaccine associated myositis (40, 41). Vaccines against hepatitis B, influenza, tetanus toxoid, H1N1 and BCG are all thought to have a temporal association with DM (42).

Whereas, the latency between COVID-19 vaccination and development of myositis symptoms varied, over half the cases had symptoms within 2 weeks of the first dose. Indeed, symptom onset occurred within 1 week for most of the patients. This represents a similar latency period to that observed with other vaccine induced myositis (42).

The majority of cases followed BNT162b2 or ChAdOx1 vaccinations, in agreement with previous reports. A study conducted in Germany evaluated neurological autoimmune diseases following COVID-19 vaccinations in which 20 of 21 patients were vaccinated with BNT162b2 (*n* = 12) or ChAdOx1 (*n* = 8) (26).

A range of clinical IM subtypes, such as DM, ASS, IMNM and PM, and induction of different myositis antibodies, such as TIF1γ, SRP, NXP2, Mi-2 and Pm/Scl-75, were found to follow COVID-19 vaccination. Furthermore, subtypes and antibody types were dependent on vaccine manufacturer. These observations indicate the heterogeneity of post COVID-19 vaccine myositis.

Dodig et al. reported the case of a 55-year-old female patient who developed myalgia, chills, fatigue and vague generalized weakness the day after her first dose of BNT162b2 vaccine. By day 21, after she had received a second dose of BNT162b2 vaccine, the symptoms had worsened to progressive weakness and on day 49 she presented at an emergency department unable to walk with a serum CK level of 7,967 IU/L (23). This reminds us that if the adverse reactions to the first vaccine dose are serious, the second dose may cause further aggravation. Specialist guidance is required for those who have a serious reaction to the first vaccine dose as to whether a second dose should be recommended.

Although the COVID-19 vaccine induces myositis symptoms, such as muscle weakness and rash, fortunately, most patients improve and symptoms may be completely relieved after receiving glucocorticoids or other immunotherapy. About half of DM patient with MDA5 antibody progressed to the complication of severe ILD which led to respiratory failure and poor prognosis despite active treatment. The anti-MDA5 antibody positive form of DM is prone to RPILD with a high mortality rate (43). Whether the vaccine accelerates disease progress is unclear and requires further research.

Many COVID-19 vaccines work by inducing a T-cell-mediated immune response to a protein translated from vaccine-delivered mRNA. mRNA vaccines show a significant level of immunogenicity and strong induction of type I interferon is a distinguishing feature (44). It is noteworthy that IM patients, especially those with DM, have an increase in type I interferon-inducible genes in muscle fibers, endothelial cells, skin and peripheral blood (45).

## Limitations

Some limitations should be considered. This review acknowledges that most of the studies were case reports, and involved small sample sizes. The protocol was submitted retrospectively. This should be noted as a major limitation. Protocols should be written prospectively i.e., before the project is conducted. Due to the heterogeneity of these studies, we cannot perform further pooled analysis of their data. However, the risk of bias shows that the articles do not follow all guidelines to appropriately report the cases of IM patients followed COVID-19 vaccination. To more clearly show the relationship between myositis and COVID-19 vaccination, more studies and multi-center data should be conducted in the future.

## Conclusion

We acknowledge that temporal association does not equate with causality and extensive epidemiological studies would be required to prove causality. A thorough clinical and pathological examination of further patients is required to elucidate the association between COVID-19 vaccination and IM. However, if vaccinated individuals experience severe and continuous muscle pain and weakness, clinicians should consider vaccine-induced inflammatory myositis which may be confirmed by measuring muscle enzyme levels and myositis autoantibodies and performing muscle biopsy for a definitive diagnosis.

## Data availability statement

The original contributions presented in the study are included in the article/Supplementary material, further inquiries can be directed to the corresponding author.

## Author contributions

YD: data curation and formal analysis. YG: data curation, methodology, and writing—review and editing. All authors contributed to the article and approved the submitted version.

## Funding

This study was supported by the National High Level Hospital Clinical Research Funding (2022-NHLHCRF-YS-02).

## Conflict of interest

The authors declare that the research was conducted in the absence of any commercial or financial relationships that could be construed as a potential conflict of interest.

## Publisher's note

All claims expressed in this article are solely those of the authors and do not necessarily represent those of their affiliated organizations, or those of the publisher, the editors and the reviewers. Any product that may be evaluated in this article, or claim that may be made by its manufacturer, is not guaranteed or endorsed by the publisher.
