# Supplementary material for: Inflammatory myopathy following coronavirus disease 2019 vaccination: A systematic review
Source: Front Public Health. 2022 Oct 21;10:1007637. doi: 10.3389/fpubh.2022.1007637 (PMC9634642; doi:10.3389/fpubh.2022.1007637)
Supplement: Supplementary file 1 [file Data_Sheet_1.PDF]

Table S1: Search strategies

Database1 PUBMED

("covid 19 vaccines"[MeSH Terms] OR ("covid 19"[All Fields] AND "vaccines"[All Fields]) OR "covid 19 vaccines"[All Fields] OR "sars cov 2 vaccine"[All Fields] OR ("covid 19 vaccines"[MeSH Terms] OR ("covid 19"[All Fields] AND "vaccines"[All Fields]) OR "covid 19 vaccines"[All Fields] OR "covid 19 vaccine"[All Fields] OR (("covid 19"[All Fields] OR "covid 19"[MeSH Terms] OR "covid 19 vaccines"[All Fields] OR "covid 19 vaccines"[MeSH Terms] OR "covid 19 serotherapy"[All Fields] OR "covid 19 serotherapy"[Supplementary Concept] OR "covid 19 nucleic acid testing"[All Fields] OR "covid 19 nucleic acid testing"[MeSH Terms] OR "covid 19 serological testing"[All Fields] OR "covid 19 serological testing"[MeSH Terms] OR "covid 19 testing"[All Fields] OR "covid 19 testing"[MeSH Terms] OR "sars cov 2"[All Fields] OR "sars cov 2"[MeSH Terms] OR "severe acute respiratory syndrome coronavirus 2"[All Fields] OR "ncov"[All Fields] OR "2019 ncov"[All Fields] OR (("coronavirus"[MeSH Terms] OR "coronavirus"[All Fields] OR "cov"[All Fields]) AND 2019/11/01:3000/12/31[Date - Publication])) AND ("vaccin"[Supplementary Concept] OR "vaccin"[All Fields] OR "vaccination"[MeSH Terms] OR "vaccination"[All Fields] OR "vaccinable"[All Fields] OR "vaccinal"[All Fields] OR "vaccinate"[All Fields] OR "vaccinated"[All Fields] OR "vaccinates"[All Fields] OR "vaccinating"[All Fields] OR "vaccinations"[All Fields] OR "vaccination s"[All Fields] OR "vaccinator"[All Fields] OR "vaccinators"[All Fields] OR "vaccine s"[All Fields] OR "vaccined"[All Fields] OR "vaccines"[MeSH Terms] OR "vaccines"[All Fields] OR "vaccine"[All Fields] OR "vaccins"[All Fields]))) OR ("covid 19 vaccines"[MeSH Terms] OR ("covid 19"[All Fields] AND "vaccines"[All Fields]) OR "covid 19 vaccines"[All Fields] OR "covid 19 vaccine"[All Fields] OR (("covid 19"[All Fields] OR "covid 19"[MeSH Terms] OR "covid 19 vaccines"[All Fields] OR "covid 19 vaccines"[MeSH Terms] OR "covid 19 serotherapy"[All Fields] OR "covid 19 serotherapy"[Supplementary Concept] OR "covid 19 nucleic acid testing"[All Fields] OR "covid 19 nucleic acid testing"[MeSH Terms] OR "covid 19 serological testing"[All Fields] OR "covid 19 serological testing"[MeSH Terms] OR "covid 19 testing"[All Fields] OR "covid 19 testing"[MeSH Terms] OR "sars cov 2"[All Fields] OR "sars cov 2"[MeSH Terms] OR "severe acute respiratory syndrome coronavirus 2"[All Fields] OR "ncov"[All Fields] OR "2019 ncov"[All Fields] OR (("coronavirus"[MeSH Terms] OR "coronavirus"[All Fields] OR "cov"[All Fields]) AND 2019/11/01:3000/12/31[Date - Publication])) AND ("vaccin"[Supplementary Concept] OR "vaccin"[All Fields] OR "vaccination"[MeSH Terms] OR "vaccination"[All Fields] OR "vaccinable"[All Fields] OR "vaccinal"[All Fields] OR "vaccinate"[All Fields] OR "vaccinated"[All Fields] OR "vaccinates"[All Fields] OR "vaccinating"[All Fields] OR "vaccinations"[All Fields] OR "vaccination s"[All Fields] OR "vaccinator"[All Fields] OR "vaccinators"[All Fields] OR "vaccine s"[All Fields] OR "vaccined"[All Fields] OR "vaccines"[MeSH Terms] OR "vaccines"[All Fields] OR "vaccine"[All Fields] OR "vaccins"[All Fields])))) AND ("polymyositis"[MeSH Terms] OR "polymyositis"[All Fields] OR ("myositis"[MeSH Terms] OR "myositis"[All Fields] OR "myositides"[All Fields] OR ("muscular diseases"[MeSH Terms] OR ("muscular"[All Fields] AND "diseases"[All Fields]) OR "muscular diseases"[All Fields] OR "myopathies"[All Fields] OR "myopathy"[All Fields])) OR ("dermatomyositis"[MeSH Terms] OR "dermatomyositis"[All Fields]))

Database2 EMBASE

#1 'covid 19 vaccine'/exp

#2 'covid 19 vaccine' OR (('covid'/exp OR covid) AND 19 AND vaccine):ab,ti

#3 'sars cov 2 vaccine':ab,ti

#4 #1 OR #2 OR #3

#5 'myositis'/exp

#6 'myositis':ab,ti

#7 'dermatomyositis':ab,ti

#8 'inflammatory myopathy':ab,ti

#9 #5 OR #6 OR #7 OR #8

#10 #4 AND #9
