# Supplementary material for: Inflammatory myopathy following coronavirus disease 2019 vaccination: A systematic review
Source: Front Public Health. 2022 Oct 21;10:1007637. doi: 10.3389/fpubh.2022.1007637 (PMC9634642; doi:10.3389/fpubh.2022.1007637)
Supplement: Supplementary file 2 [file Data_Sheet_2.pdf]

Data extraction

1. General Information	
Record title<br>First few characters of primary study author's name	
Person extracting	
Date<br>Of study publication	
Study Title	
Authors<br>Including lead author contact details	
Possible conflicts of interest	

2. Study method/characteristics	
Design<br>Cohort – prospective or retrospective	
Setting<br>May refer to hospital/community, inpatient/outpatient, rural/urban etc.	
Location<br>Country and region	
Participants<br>age (range; mean),<br>gender (male n, %; female n, %),<br>any other relevant characteristics	
Exposure(s)<br>Latency from vaccine to myositis symptoms, which type of vaccine,	

Co-morbidities	
Recruitment procedures<br>Including inclusion/exclusion criteria	
Details of administration<br>Any details on the duration of the stu dy, follow-up	
Details of participants leaving study<br>Characteristics of those	

3. Primary Outcome	
Myositis after COVID-19 vaccine (syptoms, biospy, antibodies, diagnosis, treatment)	
Outcome	

4. Secondary Aims	
Did paper assess predictors of myositis?	

What were the findings?<br>Description for results	

5. Quality Assessment	
JBI Cohort Study Checklist score	
