# Supplementary material for: Inflammatory myopathy following coronavirus disease 2019 vaccination: A systematic review
Source: Front Public Health. 2022 Oct 21;10:1007637. doi: 10.3389/fpubh.2022.1007637 (PMC9634642; doi:10.3389/fpubh.2022.1007637)
Supplement: Supplementary file 3 [file Data_Sheet_3.PDF]

S3. JBI checklists

We assessed methodological quality using the Joanna Briggs Institute checklist for Cross-Sectional Studies (Joanna Briggs Institute, 2020) with minor amendments to address the relationship to our study question.

Study	1	2	3	4	5	6	7	8	Quality judgement
Tan et al.	Y	U	U	U	U	U	U	Y	Poor
Kim et al.	Y	U	Y	Y	N	Y	U	Y	Medium
Gupta et al.	Y	U	N	N	N	Y	N	Y	Poor
Wu et al.	Y	U	Y	U	U	U	U	Y	Poor
Farooq et al.	Y	U	Y	Y	N	U	N	Y	Medium
Theodorou et al.	Y	U	Y	N	N	U	N	U	Poor

Gouda et al.	Y	Y	N	N	N	Y	N	Y	Medium
Venkateswaran et al.	Y	U	U	N	N	Y	N	U	Poor
Camargo et al.	Y	Y	Y	N	N	U	N	U	Poor
Lee et al.	Y	N	Y	N	N	N	Y	Y	Medium
Dodig et al.	Y	N	N	N	N	N	Y	Y	Poor
Vutipongsatorn et al.	Y	U	N	N	N	Y	Y	Y	Medium
Maramattom et al.	Y	U	Y	N	N	Y	Y	Y	Medium
Kaulen et al.	Y	U	U	N	N	Y	N	U	Poor
Blaise et al.	Y	Y	N	N	N	Y	Y	Y	Medium
Borio et al.	Y	N	Y	N	N	U	Y	Y	Medium

Yoshida et al.	Y	Y	N	N	N	U	N	Y	Poor
Capassoni et al.	Y	Y	N	N	N	U	Y	N	Poor
Kreuter et al.	U	Y	N	N	U	N	Y	N	Poor
Ooi et al.	Y	N	N	Y	N	N	U	N	Poor
Gouveia et al.	Y	U	N	N	Y	N	N	Y	Poor
Carrasco et al.	N	N	U	N	Y	Y	Y	U	Poor
Gonzalez et al.	U	Y	Y	U	N	Y	U	Y	Medium
Kitajima et al.	Y	Y	Y	N	U	N	Y	U	Medium
